# Biobehavioral correlates of an fMRI index of striatal tissue iron in depressed patients

**DOI:** 10.1038/s41398-021-01553-x

**Published:** 2021-09-01

**Authors:** Rebecca B. Price, Brenden C. Tervo-Clemmens, Benjamin Panny, Michelle Degutis, Angela Griffo, Mary Woody

**Affiliations:** 1 Department of Psychiatry, University of Pittsburgh, Pittsburgh, PA USA; 2 Department of Psychology, University of Pittsburgh, Pittsburgh, PA USA; 3 Department of Psychiatry, Massachusetts General Hospital, Harvard Medical School, Boston, MA USA; 4 Heinz College of Information Systems and Public Policy, Carnegie Mellon University, Pittsburgh, PA USA

**Keywords:** Depression, Human behaviour

## Abstract

Dopaminergic function is a critical transdiagnostic neurophysiological dimension with broad relevance in psychiatry. Normalized T2*-weighted (nT2*w) imaging has been previously investigated as a method to quantify biological properties of tissue in the striatum (e.g., tissue iron), providing a widely available, in vivo marker with potential relevance to dopaminergic function; but no prior study to our knowledge has examined this neuroimaging marker in clinical depression. In a treatment-seeking, clinically depressed sample (*n* = 110), we quantified tissue iron (nT2*w) in striatal regions. We assessed test-retest reliability and correlated values with dimensional features across levels of analysis, including demographic/biological (sex, age, Body Mass Index), neuroanatomical (hippocampal atrophy, which was quantified using a recently validated machine-learning algorithm), and performance-based (Affective Go/NoGo task performance) indices with relevance to depressive neurocognition. Across patients, decreased tissue iron concentration (as indexed by higher nT2*w) in striatal regions correlated with indices of decreased cognitive-affective function on the Affective Go/NoGo task. Greater caudate nT2*w also correlated with greater hippocampal atrophy. Striatal tissue iron concentrations were robustly lower in female patients than males but gender differences did not explain relations with other neurocognitive variables. A widely available fMRI index of striatal tissue properties, which exhibited strong psychometric properties and can be readily quantified from most fMRI datasets irrespective of study-specific features such as task design, showed relevance to multiple biobehavioral markers of pathophysiology in the context of moderate-to-severe, treatment-resistant depression. Striatal tissue iron may play a role in dimensional and subgroup-specific features of depression, with implications for future research on depression heterogeneity.

## Introduction

Within the field of psychiatric neuroimaging, there is a recognized need to address historically small sample sizes through the development of neuroimaging indices that demonstrate good psychometric properties and can be readily harmonized and compiled across a large number of existing and future collaborative datasets [1–3]. Resting-state protocols and harmonized imaging tasks (e.g., PhenX Toolkit) have been proposed as possible solutions. A complementary strategy is to identify novel analytic methods that can be readily applied to most fMRI datasets, irrespective of study-specific features (e.g., task design and acquisition parameters), to derive a psychometrically reliable, meaningful, and clinically relevant marker of pathophysiology.

Normalized T2*-weighted (nT2*w) imaging represents one such readily available neuroimaging marker with possible broad transdiagnostic relevance in psychiatry. T2*-weighted signal is typically examined as a time-series within an fMRI session, to assess relatively small fluctuations in signal strength that reflect blood-oxygen-level-dependent response but can also be normalized and averaged to generate a single, overall signal strength value for each individual [4, 5]. Because tissue iron is paramagnetic and disrupts the T2*-weighted signal, the strength of this normalized signal is inversely related to tissue iron concentration [6]. The tissue iron is critical to the dopaminergic system and strongly related to dopamine D2 receptors and dopamine transporter (DAT) densities, as well as the regulation and function of dopamine neurons [7, 8]. As such, it is found in high concentrations in the dopamine-rich striatal areas of the reward system, and has shown deficient levels in conditions characterized by dopaminergic dysfunction [e.g., ADHD [9]]. Previous work has suggested that when applied in the iron-rich striatal regions of the brain’s dopaminergic reward system, nT2*w markers may be related to more specialized imaging measures of tissue iron (e.g., quantitative susceptibility mapping; QSM) [10]. A related MRI index of tissue iron (R2’) has been previously related to PET markers of striatal dopamine [11], in at least some striatal areas (i.e., the nucleus accumbens, though not in dorsal striatal areas), further suggesting that dopamine neurobiology can be indexed with noninvasive MRI measures of striatal tissue iron. Furthermore, striatal nT2*w 1) follows an expected longitudinal trajectory of neurodevelopment across adolescence [5, 10] and two) tracks with performance-based behavioral indices in healthy individuals [4]. Thus, preliminary evidence supports its possible use as a widely available, in vivo marker with potential relevance to the dopaminergic function. However, few prior studies have tested whether this index is a relevant marker of psychiatric pathophysiology, and no published study to our knowledge has related this index to other markers of dysfunction in the context of depression—a psychiatric condition strongly characterized by the dopaminergic and reward system aberrations [12].

Dimensional approaches to the study of depression have recently come into prominence as a way of parsing the highly heterogeneous mechanisms, etiological factors, and clinical trajectories that characterize the diverse set of patients meeting diagnostic criteria for a depressive disorder [13]. Dimensional features of depression can be expressed at multiple levels of analysis (e.g., in behavioral patterns, structural neuroanatomy, and markers of neural function) and to the extent that such markers reflect a relatively unitary mechanism manifested across levels of analysis, correlated with one another using an integrative approach [14].

At the behavioral level, depression is characterized by notable impairments of cognitive-affective processing in performance-based measures of affective information processing. Cognitive flexibility in the processing of affective stimuli (e.g., emotional faces)—a key substrate of optimized goal-directed behavior—relies on the dopaminergic function in dorsal and ventral striatal areas [15], in coordination with prefrontal executive and attentional networks and stimulus-driven processing within cortico-mesolimbic circuits [16]. Inhibitory control of action in response to positive and/or negative stimuli (e.g., flexibly inhibiting responses to affective stimuli) is supported as a core cognitive deficit in depression [17] and is at least partially reliant on striatal circuits [18]. Thus, to the extent that striatal nT2*w signal reflects a behaviorally relevant marker of functioning within key depression-relevant mesocorticolimbic circuits, cognitive-affective flexibility impairments on task-based indices may be expected to track with individual differences in nT2*w.

In addition to the dopaminergic system changes, at the neuroanatomical level, a robust literature of both preclinical and clinical data strongly links depressive pathophysiology to hippocampal atrophy [16, 19]—which in turn has been tied to a range of cognitive impairments seen in depression [14], as well as to particularly poor clinical outcomes, e.g., completed suicide [20]. This hippocampal atrophy is posited to be the result of chronic stress exposure leading to sustained decreases in neuroprotective factors [e.g., brain-derived neurotrophic factor (BDNF) expression and signaling] that damage or hinder plasticity, fostering neuronal atrophy and decreased synaptic number and function, particularly in medial PFC and hippocampus [21, 22]. Cascading stress-related neuroplasticity changes also adversely impact striatal dopaminergic system regulation and function [23, 24]. Thus, individual differences in the degree of hippocampal atrophy exhibited by a given patient might also be expected to correlate with other ostensible markers of stress-induced neuronal and molecular dysfunction, including striatal nT2*w signal.

In the current proof-of-principle study, we used an fMRI dataset collected from 110 treatment-seeking adults with moderate-to-severe, treatment-resistant depression to quantify tissue iron concentration as nT2*w values. To aid in the consideration of nT2*w as a potential neuroimaging index with strong potential to be harmonized across samples, we aimed to quantify the psychometric test-retest reliability of this measure in a patient sample and to establish whether this index was related to other markers of neurocognitive dysfunction that were available in the context of a well-characterized depression sample. We posited that striatal nT2*w signal, as an indirect but readily available potential proxy for dopaminergic function in striatal circuits, would track dimensionally with discrete behavioral and neuroanatomical deficits in depression, helping to tie stress-related neuronal and molecular changes in key depression-relevant circuits to clinically relevant behavioral outputs.

## Methods

All data were acquired in the context of an ongoing randomized controlled trial (R01MH113857; clinicaltrials.gov: NCT03237286). In brief, 110 patients reporting moderate-to-severe levels of depression [Montgomery-Asberg Depression Rating Scale (MADRS; [25]) score ≥ 25] and at least one failed, an adequate trial of an FDA-approved antidepressant medication in the current depressive episode were recruited for an experimental therapeutics study and completed a neuroimaging assessment prior to commencing any study intervention procedures. Any existing depression treatment regimens were stably maintained throughout the study period. See Table 1 and Supplement for sample characteristics and details of baseline clinical assessments. The study was performed at the University of Pittsburgh and approved by the Internal Review Board of the University of Pittsburgh. All participants provided informed consent prior to any study procedure.

**Table 1:** Demographic and clinical characteristics of the sample.

	*N* = 110
Race:	
Caucasian, *n* (%)	92 (84%)
Black, *n* (%)	4 (4%)
Asian, *n* (%)	6 (6%)
Multiracial, *n* (%)	7 (6%)
Unknown, *n* (%)	1 (1%)
Ethnicity:	
Non-Hispanic, *n* (%)	97 (88%)
Hispanic/Latino, *n* (%)	8 (8%)
Unknown, *n* (%)	5 (5%)
Female sex at birth, *n* (%)	69 (63%)
Gender:	
Cisgender male	40 (36%)
Cisgender female	62 (56%)
Transgender: female-to-male	1 (0.9%)
Nonbinary or gender fluid	2 (1.8%)
Gender undisclosed/unknown	5 (5%)
Age	35.2 (11.1)
MADRS score	34.6 (4.7)

*MADRS* Montgomery-Asberg Depression Rating Scale.

Data presented as mean (SD) unless otherwise noted.

### fMRI acquisition and analysis

**Fig. 1:**
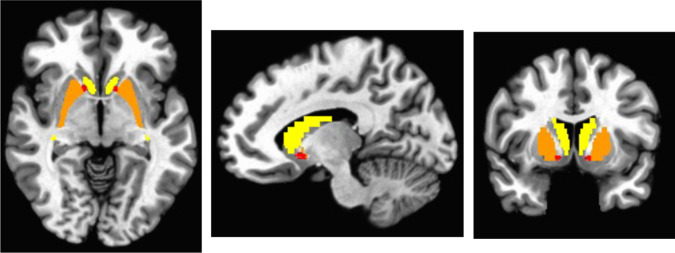
Anatomically defined regions of interest (ROIs) where striatal normalized T2*-weighted signal was extracted. Bilateral caudate shown in yellow, bilateral putamen shown in orange, bilateral nucleus accumbens shown in red.

BOLD data were acquired on a 3Tesla Siemens PRISMA scanner using Human Connectome Project sequences (multi-band factor = 8; TR = 800 ms; TE = 37; flip angle=52°; 72 slices; FOV = 200 × 200; 2 mm isotropic voxels). After standard preprocessing steps were applied in AFNI, normalized T2*-weighted (nT2*w) signal values were then computed as previously described [4, 5]. Each TR was first scaled/normalized to its own mean across the scan run (i.e., each voxel’s T2* signal value at a given TR was divided by the brain-wide average T2* signal at that TR, averaged across all voxels within a brain mask derived from the MNI template), which eliminates scan-specific variability and allows for valid comparison of T2* values across participants. We then extracted the median of this normalized signal across all TRs in the run, voxel-wise, yielding a single nT2*w map per participant. A priori striatal masks, defined anatomically via the MNI atlas (Fig. 1), were applied to extract the average normalized signal in each of the following 3 ROIs, averaged across the left and right hemispheres (to reduce multiple comparisons, as lateralized findings in the striatum were not strongly anticipated): caudate and putamen (dorsal striatum) and nucleus accumbens (ventral striatum). See Supplement for full details of fMRI data acquisition and analysis and for hemisphere-specific analyses (Table S1), which confirmed our assumption that observed patterns were largely consistent across hemispheres.

The primary nT2*w values used for analysis was derived during a 7 min resting-state block with eyes open (525 TRs) at the start of a 60 min scan session. Of note, though we selected resting-state data as our primary index to its wide availibility, nT2*w is expected to be fully impervious to task condition/state and applicable equally to any fMRI dataset—an assumption that we tested here by calculating test-retest reliability across resting-state and task-based (positive mood induction) data. To quantify test-retest reliability (intraclass correlation coefficient; ICC), identical methods were applied separately (including separate application of all preprocessing steps, e.g., independent registration to the structural scan, warping to the MNI template, etc.) to derive nT2*w values during two retest sets: 1) a second 7 min block of fMRI data collected during a positive mood induction task collected at the end of the 60 min scan session (short-term test-retest reliability); and 2) during an identical 7 min resting-state block collected at a 1-week retest interval (in *n* = 101 patients; 1-week test-retest reliability).

### Affective Go/NoGo task

This task was collected in the current study as the primary behavioral index of cognitive-affective flexibility. As in prior work [26], patients were instructed to press a button when a specific facial expression target was displayed on a computer screen (i.e., happy, sad, and neutral). During each 500 ms face presentation, patients were asked to press a button as fast as possible when the target expression was presented (i.e., on “Go” trials, 70% of trials) but to withhold pressing a button for the “NoGo” facial expression. During each block, an emotional expression (happy or sad) was always paired with a neutral expression, and depending on the block, either the emotional expression served as the “Go” stimulus or as the “NoGo” stimulus. Following practice trials, there were four randomized blocks (Go = sad/NoGo = neutral, Go = neutral/NoGo = sad, Go = happy/NoGo = neutral, and Go = neutral/NoGo = happy) each with 30 trials presented in random order. False alarm rate (i.e., an error of commission by pressing the button during a “NoGo” trial) was calculated as the proportion of total false alarms to total “NoGo” trials. Hit rate was calculated as the proportion of correct responses to total “Go” trials. To index emotion discrimination, *“*d-prime” was calculated by subtracting the *z*-transformed false alarm rate from the *z*-transformed hit rate (*z*-transformations based on the sample mean and SD, calculated independently for each variable, such that, prior to subtraction, the sample distributions for each variable were centered around 0 with SD = 1). Overall false alarm rate, inverted (subtracted from 0) such that larger values indicate better performance (for consistency with other metrics), served as an index of general cognitive control, and inverted false alarm rate specifically to emotional “NoGo” stimuli served as an index of emotion regulation.

### Hippocampal parenchymal fraction (HPF)

To quantify degree of hippocampal atrophy in the present dataset, the structural MPRAGE image collected in the MRI session was passed to a publicly available, machine-learning-based algorithm (KAIBA; https://www.nitrc.org/frs/shownotes.php?release_id=3733) that has been described in detail previously [27]. In brief, KAIBA estimates the expected hippocampal volumetric morphometry for each individual based on >100 automatically detected neuroanatomical landmarks throughout the rest of their brain, and then compares the expected hippocampal volume to its observed volume to derive a Hippocampal Parenchymal Fraction (HPF) in each hemisphere (higher HPF values = less atrophy). This approach has been previously shown to be more sensitive than hippocampal volume (e.g., as estimated using the FreeSurfer software) in separating between Alzheimer’s disease patients and healthy age-matched controls [27], is a sensitive biomarker of progression from mild cognitive impairment to Alzheimer’s disease [28], and can be computed in a small fraction of the time required by other volumetric analysis software with minimal data loss due to failures to segment properly, etc. Because asymmetries within left-vs-right hippocampal atrophy are potentially clinically relevant features in some psychiatric populations [29, 30], we analyzed left and right HPF separately.

### Demographic/biological features

Based on prior reported relationships to tissue iron [10, 31, 32], we examined whether nT2*w was related in our sample to biological birth sex (as listed on patients’ birth certificates), age, or BMI in bivariate relationships. In sensitivity analyses, we examined whether controlling for these variables altered other primary findings.

### Analyses

For continuous variables, pairwise correlations with nT2*w signal in striatal ROIs were computed and corrected for multiple comparisons within each domain via the widely-used and well-validated False Discovery Rate (FDR) correction method [33]; for completeness, both the corrected and uncorrected (two-tailed) *p* values are reported. For the categorical variable we examined (sex), unpaired *t* tests were used to interrogate differences within each ROI separately, with multiple comparisons correction applied across the three ROIs using the FDR method. Assumptions of normality and equal variance were upheld.

## Results

### Reliability

Test-retest correlations of nT2*w signal in each ROI were examined across a ~50 min interval using two 7 min blocks of fMRI data collected at the beginning and end of a single scan session (short-term reliability), as well as at a 1-week interval. Test-retest reliability was excellent by conventional psychometric standards (short-term—caudate: ICC = 0.93; putamen: ICC = 0.90; nucleus accumbens: ICC = 0.91; 1-week—caudate: ICC = 0.84; putamen: ICC = 0.82; nucleus accumbens: ICC = 0.80; all *p*’s < 0.001).

### Correlates

#### Go/NoGo performance

**Fig. 2:**
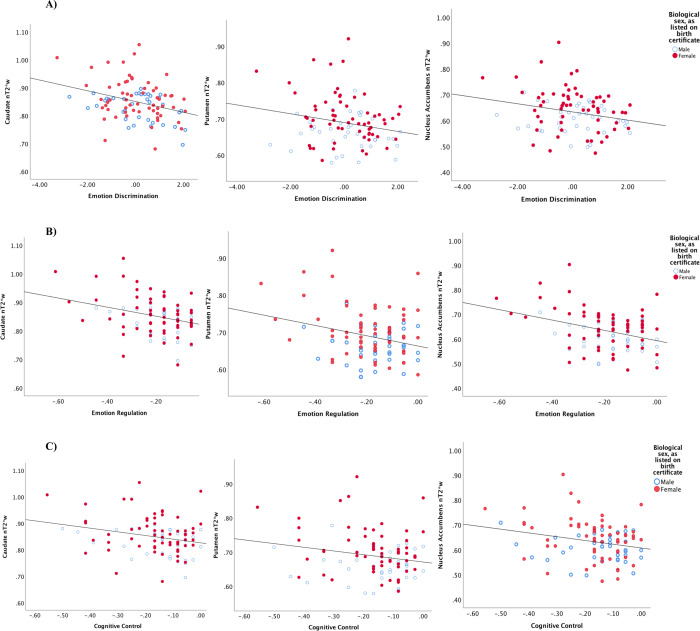
Scatterplots depicting the nature of correlational relationships between striatal normalized T2*-weighted (nT2*w) signal and Go-NoGo performance-based indices across patients, as reported in Table 2. For nT2*w signal, higher values indicate lower tissue iron concentration; for task indices, higher values indicate better performance. Sex differences in nT2*w are simultaneously illustrated through individual points for female (red, filled) and male (blue, hollow) participants. Scatterplots depict nT2*w correlational relationships with performance-based indices of **A** emotion discrimination; **B** emotion regulation; and **C** cognitive control.

Performance-based indices of emotion discrimination, general cognitive control, and emotion regulation were related to nT2*w across striatal regions in the expected directions across every pairwise ROI-task (|*r* | ≥.20; *p*_uncorrected _*=* 0.00038 − 0.046; *p*_corrected _= 0.003 − 0.046; Table 2; Fig. 2), suggesting greater striatal tissue iron concentration tracked robustly with greater cognitive-affective flexibility during the task.

**Table 2:** Correlation coefficients and statistics for relationships of striatal normalized T2*-weighted signal with biobehavioral variables in the study sample.

Neurocognitive index	Striatal normalized T2*-weighted signal		
	Caudate	Putamen	Nucleus accumbens
**Go/NoGo task** Emotion discrimination	−**0.33 (*p*** = **0.001;** ***R***^**2** ^**=** **0.11)**	−**0.21 (*p*** = **0.036;** ***R***^**2** ^**=** **0.04)**	−**0.23 (*p*** = **0.020;** ***R***^**2** ^**=** **0.05)**
General cognitive control	−**0.25 (*p*** = **0.012;** ***R***^**2** ^**=** **0.06)**	−**0.20 (*p*** = **0.046;** ***R***^**2** ^**=** **0.04)**	−**0.24 (*p*** = **.017;** ***R***^**2** ^**=** **0.06)**
Emotion regulation	−**0.30 (*p*** = **0.002;** ***R***^**2** ^**=** **0.09)**	−**0.27 (*p*** = **0.007;** ***R***^**2** ^**=** **0.07)**	−**0.35 (*p*** ***<*** **.001;** ***R***^**2** ^**=** **0.12)**
**HPF** Right hippocampus	−**0.26 (*p*** = **0.007;** ***R***^**2** ^**=** **0.07)**	0.01 (*p* = 0.959; *R*^2 ^< 0.001)	0.04 (*p* = .656; *R*^2 ^= 0.001)
Left hippocampus	−*0.19 (p* *=* *0.047; R*^*2* ^*=* *0.04)*	0.03 (*p* = 0.734; *R*^2 ^< 0.001)	0.06 (*p* = 0.557; *R*^2 ^= 0.004)

Pearson’s r correlation coefficients and associated uncorrected p-values. Bold text indicates correlation is significant at the 0.05 level (2-tailed) after correcting for multiple comparisons; italicized text indicates significant at the 0.05 level in uncorrected analyses only. Go/NoGo task indices coded such that higher values indicate better task performance. HPF = Hippocampal Parachymal Fraction, higher scores indicate greater hippocampal volumetric integrity (less atrophy). For normalized T2*-weighted signal, higher values indicate lower tissue iron concentration.

#### Hippocampal volumetric integrity (HVI)

**Fig. 3:**
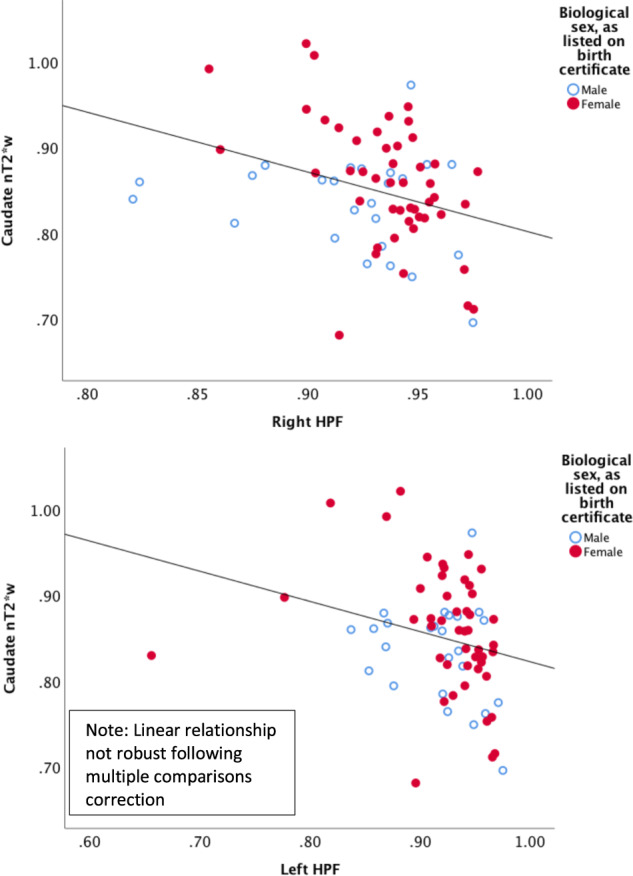
Scatterplots depicting the nature of correlational relationships between normalized T2*-weighted (nT2*w) signal in the caudate and Hippocampal Parachymal Fraction (HPF) in the right and left hippocampus, as reported in Table 2. For nT2*w signal, higher values indicate lower tissue iron concentration; for HPF, higher values indicate increased hippocampal integrity (e.g., less atrophy). Sex differences in nT2*w are simultaneously illustrated through individual points for female (red, filled) and male (blue, hollow) participants.

Lesser nT2*w signal (indicating greater tissue iron concentration) in the caudate correlated with greater HVI in the right (*r* = −0.19; *p*_uncorrected _= 0.007; *p*_corrected _= .042) and left (*r* = −0.26; *p*_uncorrected _= 0.047; *p*_corrected _= 0.12) hippocampus, although only the right hemisphere finding was robust to multiple comparisons correction (Table 2; Fig. 3).

#### Clinical and demographic features

Across striatal regions, nT2*w signal was robustly related to sex with medium-to-large effect sizes (males < females; putamen: *p*_uncorrected _< 0.001, *p*_corrected _< 0.001; nucleus accumbens: *p*_uncorrected _< 0.001, *p*_corrected _= 0.001; caudate: *p*_uncorrected _= 0.024, *p*_corrected _= 0.024; Cohen’s d = 0.43−0.74; Figs. 2–3**)**. nT2*w was related to BMI only in the nucleus accumbens (*r* = 0.31*; p*_uncorrected _= 0.001; *p*_corrected _= 0.003), and to age only in the putamen (and only in uncorrected analyses; *r* = −0.19*; p*_uncorrected _= 0.04; *p*_corrected _= 0.12). No regions were related to psychotropic medication status or overall depression severity (Montgomery-Asberg Depression Rating Scale) scores (*p*_uncorrected_’s > 0.15). Significant findings described above were upheld in partial correlations controlling for sex, BMI, and age; see Table S2 and Supplement for details.

## Discussion

Dopaminergic function within neural circuits that include the dorsal and ventral striatal regions is a key substrate of multiple aspects of cognition with high relevance to depression [15], including altered inhibition and inflexible processing of positive and negative affective stimuli [17, 34]. Most methods for probing dopaminergic function in human patients rely on specialized procedures and equipment, which are not readily available in large imaging corpi and present technical challenges for large-scale data collection. Here, we used a very widely available marker of tissue properties indexing iron concentration within striatal regions as a possible novel proxy marker of dopaminergic function among treatment-seeking depressed patients. We showed that this novel marker demonstrated excellent test-retest reliability (at both 50-min and 1-week test-retest intervals) and was linked to individual differences across two discrete domains that we hypothesized would be mechanistically linked to striatal dopaminergic function: performance-based measures of cognitive-affective flexibility and structural measures of hippocampal atrophy. Consistent with previous reports in healthy individuals [10, 31], a sexual dimorphism in tissue iron was also observed, which was strongly evident across dorsal and ventral striatum, yet independent of the relationships observed in other levels of analysis. This readily available avenue for fMRI data analysis might thus help generate novel insights regarding the nature and mechanisms of clinical depression.

Impairments in cognitive-affective flexibility and inhibition of affective stimuli among depressed patients are evident across a range of performance-based measures [17, 34, 35], including decreased affective flexibility indexed by the Affective Go/NoGo task [36]. Performance of this and similar tasks requiring flexible inhibition of responses to affective stimuli has been previously linked to altered reactivity and function of striatal, prefrontal, and amygdalar regions within key depression-relevant dopaminergic (e.g., cortico-mesolimbic) circuits [37–41], though few neuroimaging studies of depressed patients have examined the substrates of these patterns. The present work suggests the novel finding that these behavioral patterns—including both the emotion-specific inhibitory and discrimination deficits, as well as more generalized cognitive control decrements—are tied to decreased tissue iron concentrations, widely distributed across the dorsal and ventral striatum. Though further validation work of nT2*w as a possible marker of dopaminergic function is needed, such decreases could reflect the decreased proliferation of key dopamine system constituents (D2 receptors; DAT) [7, 8] that collectively reduce the brain’s capacity to flexibly integrate and transmit inhibitory behavioral signals within dopaminergic mesocorticolimbic circuits.

Neuroplasticity impairments, such as neuronal atrophy, decreased synaptic number and function, and decreases in neuroprotective factors [e.g., brain-derived neurotrophic factor (BDNF) expression and signaling], evident most prominently in the hippocampus and medial PFC, are strongly supported molecular substrates of depressive-like behavior in rodents exposed to chronic stress [16]. Convergent evidence from postmortem and volumetric analysis of the brain structure in depressed patients [16] suggests hippocampal atrophy is a highly reliable biomarker of depression risk [19], and is linked to severe outcomes such as completed suicide [20]. Hippocampal atrophy is particularly robust in patients with recurrent and early-onset forms of depression [19], suggesting the impact of the depressed state on hippocampal volume may be cumulative across time and/or episodes. Striatal areas are likewise reshaped at the neuronal and molecular levels by chronic stress [23, 24], suggesting a cascading impact of stress on plasticity and neuronal function that impacts multiple depression-relevant circuits in tandem [14]. Using a novel, recently validated method to automatically quantify hippocampal volumetric integrity based on machine-learning algorithms [27], we found that greater caudate tissue iron concentration among depressed patients was also related to greater hippocampal integrity, with particularly robust findings for the right hippocampus. The caudate nucleus has direct and indirect projections to the hippocampus, supporting numerous aspects of memory and learning [15]. Thus, the combined insult of decreased caudate tissue iron and hippocampal atrophy observed in some depressed patients in our sample might contribute to the generalized memory and cognitive impairments also widely observed in depression [42, 43]—a hypothesis that could be readily tested in the future work using the novel neuroimaging methods described here.

Striatal nT2*w was also robustly linked to self-reported biological sex (as listed on one’s birth certificate), with female patients showing evidence of decreased tissue iron concentration relative to males. This sex difference was medium-to-large, evident across dorsal, and ventral striatum, and yet it did not appear to explain the dimensional findings observed in other domains. Some prior reports in healthy samples have likewise reported gender differences in striatal tissue iron, though the direction of effect may vary across the course of development [10, 31]. Sex discrepancies previously reported in the context of depression include the increased overall rate of depression among females, which rises to 2x the rate in males beginning in adolescence; and higher rates in females of negative rumination [44], “atypical” symptoms (e.g., increased appetite, increased sleep), comorbid anxiety, and suicide attempts (but not suicide completions) [45]. Gene expression in postmortem tissue exhibits strikingly divergent molecular transcriptional signatures across male- and female- depressed patients [46], with wide-ranging implications for potentially divergent disorder mechanisms. We previously reported that two data-driven subgroups of depression, defined purely on the basis of resting-state connectivity patterns across key prefrontal and mesolimbic regions (including nucleus accumbens), exhibited a sexual dimorphism in terms of subgroup frequency [47]—although females were well-represented in both subgroups, suggesting within-sex heterogeneity is also prominent. Overall, few studies have examined the question of whether the heterogeneous neurobiological substrates of depression may affect males and females at differing rates, which could implicate discrete treatment targets. Our findings add to a growing literature suggesting further well-powered studies are warranted.

## Limitations

The present proof-of-concept analyses focused exclusively on individual differences among moderate-to-severely depressed patients. While this dimensional approach acknowledges the substantial heterogeneity that is strongly characteristic of treatment-seeking depressed patients and supports neurophysiological relevance within a richly phenotyped, treatment-resistant sample, findings may not generalize to healthy individuals, those with lower levels of depression, or those with remitted or prodromal forms of depression. Of note, though nT2*w signal was not related to depression severity, depression severity was characterized by a restricted range of values given that all enrolled patients were required to have moderate or greater depression scores, thus limiting capacity to test these relationships in the current study. Furthermore, effect sizes for correlations were generally small-to-medium, suggesting significant variance in measured constructs remained unexplained. This likely reflects multiple diverse contributors to biological, behavioral, and clinical patterns in human patients, with small effects reflecting inherent and meaningful complexity of psychological phenomena [48], in addition to measurement error. nT2*w signal is an indirect measure of both the tissue iron concentration and, potentially, dopaminergic function, though it has been previously shown to correlate with a more direct neuroimaging measure of tissue iron [10]. Our performance-based indices of cognitive function were derived from a single task used to index cognitive-affective flexibility within the scope of an ongoing RCT. Additional performance-based tasks, e.g., those that directly probe reward function, would extend these initial findings to other prominent cognitive impairments that have been closely tied to striatal dopaminergic function. Similarly, the novel machine-learning algorithm we used to quantify hippocampal atrophy is not currently applicable to other regions of the brain. While our sample did include some individuals whose gender identity was divergent from their biological sex at birth (Table 1), we focused exclusively on biological sex as we were underpowered to conduct a thorough analysis of gender as a discrete construct. Finally, the study consisted of predominantly non-Latinx White patients, which although representative of the levels of racial and ethnic diversity within the geographic county where the study was performed, is not representative of the general population in the United States. Care must be taken in future research to recruit diverse samples that will allow researchers to better characterize the relation between nT2*w and other clinically relevant outcomes among underserved populations that have historically been underrepresented in psychiatric research.

## Conclusions

Striatal nT2*w signal, which prior work has suggested correlates closely with more specific in vivo assays of tissue iron concentration, can be easily quantified from virtually any existing fMRI dataset, making it an attractive choice for future mechanistic work in depressed patients. The strong psychometric properties we report here are critical to overcoming a major limitation of many neuroimaging indices which threatens to cripple progress—particularly for analyses that rely heavily on accurate, patient-level quantification, as is necessary for the development of precision medicine tools (e.g., clinical decision algorithms) and biomarkers of treatment outcome [1]. The current analyses provide preliminary validation of this measure’s relevance to other, clinically relevant dimensions of neurocognitive dysfunction among treatment-seeking patients with moderate-to-severe, treatment-resistant depression—a population at high risk of poor psychiatric outcomes [49]. Robust correlations were observed that linked nT2*w signal to both the behavioral and neuroanatomical levels of analysis, suggesting it represented a clinically relevant marker of dysfunction within the mesolimbic and affective circuits that are influential and well-established in depressive pathophysiology. Future neuroimaging analyses may benefit from the inclusion of this readily attainable index to help triangulate mechanistic targets for depression prevention and treatment.

## Supplementary information

Supplement

## Data Availability

The code used to generate nT2w* values and statistical tests is available from the first author (RBP) upon request.
