# Supplementary material for: Biobehavioral correlates of an fMRI index of striatal tissue iron in depressed patients
Source: Transl Psychiatry. 2021 Sep 1;11:448. doi: 10.1038/s41398-021-01553-x (PMC8410762; doi:10.1038/s41398-021-01553-x)
Supplement: Supplementary file 1 — Supplement [file 41398_2021_1553_MOESM1_ESM.docx]

**Biobehavioral Correlates of an fMRI Index of Striatal Tissue Iron in Depressed Patients**

***Supplemental Information***

[Supplemental Methods & Materials 2](#_Toc79055451)

[Participants 2](#_Toc79055452)

[Power analysis 4](#_Toc79055453)

[fMRI preprocessing and analysis 4](#_Toc79055454)

[Missing Data and Data Cleaning 5](#_Toc79055455)

[Supplemental Analyses 7](#_Toc79055456)

[Table S1: Correlations for right- and left-hemisphere striatal nT2*-weighted signal reveal consistency in correlational patterns across hemispheres 7](#_Toc79055457)

[Table S2: Partial correlations between striatal T2* signal and neurocognitive indices, controlling for sex, age, and BMI 8](#_Toc79055458)

# Supplemental Methods & Materials

## Participants

Participants were recruited and enrolled from 12/01/2017 to 12/01/2020. Inclusion criteria for the larger, ongoing clinical trial (clinicaltrials.gov: NCT03237286) specified that participants: 1) be between the ages of 18 and 60 years; 2) have not responded to one or more adequate trials of FDA-approved antidepressants within the current depressive episode, determined by Antidepressant Treatment History Form; 3) score ≥ 25 on the Montgomery Asberg Depression Rating Scale (MADRS); 4) score >1SD above the normative mean on the Cognitive Triad Inventory "self" subscale *OR* <1SD below the normative mean on the Rosenberg self-esteem scale; 5) possess a level of understanding sufficient to agree to all tests and examinations required by the protocol and must sign an informed consent document; and 6) agree to sign a release of information (ROI), identifying another individual [friend, family member, etc.] as a contact person while the patient is enrolled in the study. **Table 1** in the main text presents clinical and demographic information on the sample. Diagnoses were established by experienced master’s-level (or higher) clinicians using the MINI International Neuropsychiatric Interview.

Clinical trial exclusion criteria included the following:

1. Presence of lifetime bipolar, psychotic, or autism spectrum; current problematic substance use (e.g., substance use disorder); or lifetime recreational ketamine or PCP use
2. Use of a Monoamine Oxidase Inhibitor (MAOI) within the previous 2 weeks
3. Failure to meet standard MRI inclusion criteria: those who have cardiac pacemakers, neural pacemakers, cochlear implants, metal braces, or other non-MRI-compatible metal objects in their body, especially in the eye. Dental fillings do not present a problem. Plastic or removable dental appliances do not require exclusion. History of significant injury or surgery to the brain or spinal cord that would impair interpretation of results.
4. Current pregnancy or breastfeeding, or failure to engage in an effective birth control strategy throughout the duration of the study
5. Acute suicidality or other psychiatric crises requiring treatment escalation.
6. Changes made to treatment regimen within 4 weeks of baseline assessment
7. Reading level <6th grade
8. For study entry, patients must be reasonable medical candidates for ketamine infusion, as determined by a board-certified physician co-investigator during study screening. Serious, unstable medical illnesses including respiratory [obstructive sleep apnea, or history of difficulty with airway management during previous anesthetics], cardiovascular [including ischemic heart disease and uncontrolled hypertension], and neurologic [including history of severe head injury] will be exclusions.
9. Clinically significant abnormal findings of laboratory parameters [including urine toxicology screen for drugs of abuse], physical examination, or ECG.
10. Uncontrolled or poorly controlled hypertension, as determined by a board-certified physician co-investigator's review of vitals collected during screening and any other relevant medical history/records.
11. Patients with one or more seizures without a clear and resolved etiology.
12. Patients starting hormonal treatment (e.g., estrogen) in the 3 months prior to Screening. Birth control is not an exclusion.
13. Past intolerance or hypersensitivity to ketamine or midazolam.
14. Patients taking medications with known activity at the NMDA or AMPA glutamate receptor [e.g., riluzole, amantadine, lamotrigine, memantine, topiramate, dextromethorphan, D-cycloserine], or the muopioid receptor.
15. Patients taking any of the following medications: St John's Wort, theophylline, tramadol, metrizamide
16. Patients who have received ECT in the past 6 months prior to Screening.
17. Patients currently receiving treatment with vagus nerve stimulation (VNS) or repetitive transcranial stimulation (rTMS).

The study was performed at the University of Pittsburgh and approved by the Internal Review Board of the University of Pittsburgh. All participants provided informed consent prior to any study procedure.

## Power analysis

The available sample size for these analyses (n=110) provides adequate (80%) power to identify moderate correlations (|*r| ≥* .298) in the worst-case-scenario in which only one significant ROI after adjusting for multiple comparisons. Power using False Discovery Rate correction for multiple comparisons improves with a larger number of significant tests.

## fMRI preprocessing and analysis

Standard preprocessing steps were applied using Analysis of Functional Neuroimaging (AFNI) via the afni_proc.py pipeline. The following preprocessing steps were applied: slice time correction, 6-parameter motion correction, spatial distortion correction utilizing forward- and reverse-direction Spin Echo fieldmaps and AFNI’s “blip” step, cross-registration of functional data to a high-resolution structural scan acquired in the same fMRI session (axial MPRAGE: TR=2400; TE=2.22; 208 slices; flip angle=8°; 0.8mm isotropic voxels), 32-parameter nonlinear warping to the Montreal Neurological Institute Colin-27 brain data set, spatial smoothing [6-mm full width half maximum], scaling each voxel to its own mean (see main text).

## Missing Data and Data Cleaning

Of the 110 participants who completed the fMRI scan, n=12 were excluded from analyses of the Go/No-Go task. Participants were excluded because they did not complete the task due to time constraints (n=8) or because they failed to register a single response during one or more of the four Go/No-Go blocks (n=4).

# Supplemental Analyses

## Table S1: Correlations for right- and left-hemisphere striatal nT2*-weighted signal reveal consistency in correlational patterns across hemispheres

| Neurocognitive Index | Striatal normed T2*-weighted signal | | | | | |
| --- | --- | --- | --- | --- | --- | --- |
|  | L Caudate | R Caudate | L Putamen | R Putamen | L Nucleus Accumbens | R Nucleus Accumbens |
| **Go/NoGo Task**  Emotion discrimination | **-.29 (*p*=.003)** | **-.34 (*p*=.001)** | *-.18 (p=.082)* | **-.23 (*p*=.025)** | **-.20 (*p*=.044)** | **-.25 (*p*=.013)** |
| General Cognitive Control | **-.22 (*p*=.029)** | **-.27 (*p*=.007)** | *-.19 (p=.066)* | *-.20 (p=.050)* | **-.22 (*p*=.027)** | **-.24 (*p*=.016)** |
| Emotion Regulation | **-.28 (*p*=.005)** | **-.31 (*p*=.002)** | **-.26 (*p*=.010)** | **-.26 (*p*=.010)** | **-.35 (*p<*.001)** | **-.34 (*p=*.001)** |
| **HPF**  Right hippocampus | **-.25 (*p*=.009)** | **-.26 (*p*=.008)** | .01 (*p*=.948) | .003 (*p*=.948) | .03 (*p*=.726) | .05 (*p*=.608) |
| Left hippocampus | *-.18 (p=.063)* | *-.20 (p=.045)* | .07 (*p*=.495) | -.002 (*p*=.981) | .03 (*p*=.730) | .08 (*p*=.434) |
|  |  |  |  |  |  |  |
|  |  | |  | |  | |

Note: Pearson’s r correlation coefficients and associated uncorrected p-values. Bold text indicates correlation is significant at the 0.05 level (2-tailed) after correcting for multiple comparisons; italicized text indicates significant at the .05 level or near-significant (p<.10) in uncorrected analyses only. Go/NoGo task indices coded such that higher values indicate better task performance. HPF = Hippocampal Parachymal Fraction, higher scores indicate greater hippocampal volumetric integrity (less atrophy). For normed T2*-weighted signal, higher values indicate lower tissue iron concentration.

## Table S2: Partial correlations between striatal T2* signal and neurocognitive indices, controlling for sex, age, and BMI

| Neurocognitive Index | Striatal normed T2*-weighted signal | | |
| --- | --- | --- | --- |
|  | Caudate | Putamen | Nucleus Accumbens |
| **Go/NoGo Task**  Emotion discrimination | **-.34 (*p*=.001; R^2^=.12)** | **-.27 (*p*=.013; R^2^=.07)** | **-.30 (*p*=.006; R^2^=.09)** |
| General Cognitive Control | **-.27 (*p*=.014; R^2^=.07)** | -.18 (*p*=.106; R^2^=.04) | **-.29 (*p*=.008; R^2^=.08)** |
| Emotion Regulation | **-.27 (*p*=.014; R^2^=.09)** | -.20 (*p*=.069; R^2^=.04) | **-.34 (*p=*.002; R^2^=.12)** |
| **HPF**  Right hippocampus | **-.30 (*p*=.006; R^2^=.09)** | -.18 (*p*=.100; R^2^=.03) | -.06 (*p*=.565; R^2^=.004) |
| Left hippocampus | -.17 (p=.133; R^2^=.03) | -.04 (*p*=.742; R^2^=.002) | .10 (*p*=.355; R^2^=.01) |
|  |  |  |  |
|  |  |  |  |

Note: Results were highly convergent when repeating the above partial correlations covarying one covariate at a time. Pearson’s r correlation coefficients and associated uncorrected p-values. Bold text indicates correlation is significant at the 0.05 level (2-tailed) after correcting for multiple comparisons. Go/NoGo task indices coded such that higher values indicate better task performance. HPF = Hippocampal Parachymal Fraction, higher scores indicate greater hippocampal volumetric integrity (less atrophy). For normed T2*-weighted signal, higher values indicate lower tissue iron concentration.
